# Building Biomedical Imaging and Informatics e-Science platform for translational medical research

**DOI:** 10.1186/1479-5876-10-S2-A32

**Published:** 2012-10-17

**Authors:** Jianguo Zhang, Kai Zhang, Tusheng Wang, Yuanyuan Yang, Haibo Hu, Lisa Xu

**Affiliations:** 1 Shanghai Institute of Technical Physics, Chinese Academy of Sciences, Shanghai, China; 2 School of Biomedical Engineering and Med-X Research Institute, Shanghai Jiaotong University, Shanghai, China

## Background

As there are urgent demands to bring medical imaging research and clinical service together more closely to solve the problems related to disease discover, medical research, diagnosis and education, a new imaging and informatics infrastructure and paradigm need to be developed to promote multiple disciplines of medical researchers, clinical physicians and biomedical engineers working together in a secured, efficient, and transparent cooperative environment [1]. In this presentation, we outline our preliminary work of building Biomedical Imaging and Informatics (BMII)“e-Science” platform to support collaborative research among multi-disciplines to enable translational research in multiple affiliated hospitals and academic institutions of Shanghai Jiaotong University (SJTU), and Chinese Academy of Sciences (CAS).

## Materials and methods

SJTU has 12 large affiliated hospitals located in multiple districts of Shanghai city with a lot of medical and biomedical imaging modalities (e.g., Clinical CT/MR, Micro-PET/CT) being decentralized used in these hospitals and research centers. Also, there is a powerful Shanghai Synchronic Radiation Facility (SSRF) developed by CAS to support large scale of biomedical imaging researches from molecular level to organ parts [2]. So, we designed and developed the e-Science platform to promote the multi-disciplines working together cross these hospitals and academic institutions, and adopted the Service-Oriented Architecture and grid-based concept to build it. In order to enable efficient collaborating, we designed the work and data flows with Principal Investigator (PI)-oriented information model, and developed a documents/data sharing mechanism based on IHE XDS/XDS-I profiles and the access control standard of XACML in this platform.

## Results

We implemented the BMII e-Science platform crossing Shanghai Ruijin Hospital, two campuses of SJTU, SSRS and Shanghai Institute of Technical Physics, CAS. The data communications of the e-Science platform from site to site are fast enough as they are going through the China Education Network in Shanghai with backbone of a few of GB/sec. There were two kinds of collaborations in the e-Science platform, one is to perform real-time interactively or synchronously biomedical imaging experiment among onsite users and remote users, and the other is to share the image data or documents among collaborators.

## Conclusions

The developed BMII e-Science platform can promote multiple disciplines of medical researchers, clinical physicians and biomedical engineers working together in a secured, efficient, and transparent cooperative networking environment. Now, the researches, clinical physicians and students can use this e-Science platform to perform biomedical imaging experiments and to do collaborative researching cross multiple hospitals and academic institutions.
